# The Response Regulator RegA Is a Copper Binding Protein That Covalently Dimerizes When Exposed to Oxygen

**DOI:** 10.3390/microorganisms10050934

**Published:** 2022-04-29

**Authors:** Nijia Ke, Carl E. Bauer

**Affiliations:** Molecular and Cellular Biochemistry Department, Indiana University, Bloomington, IN 47405, USA; nijke@iu.edu

**Keywords:** two-component system, RegA, aerobic, copper ion, disulfide bond, DNA binding, gene regulation

## Abstract

In *Rhodobacter capsulatus*, the histidine kinase RegB is believed to phosphorylate its cognate transcriptional factor RegA only under anaerobic conditions. However, transcriptome evidence indicates that RegA regulates 47 genes involved in energy storage, energy production, signaling and transcription, under aerobic conditions. In this study, we provide evidence that RegA is a copper binding protein and that copper promotes the dimerization of RegA under aerobic conditions. Inductively coupled plasma mass spectrometry (ICP-MS) analysis indicates that RegA binds Cu^1+^ and Cu^2+^ in a 1:1 and 2:1 ratio, respectively. Through LC-MS/MS, ESI-MS and non-reducing SDS-PAGE gels, we show that Cu^2+^ stimulates disulfide bond formation in RegA at Cys156 in the presence of oxygen. Finally, we used DNase I footprint analysis to demonstrate that Cu^2+^-mediated covalent dimerized RegA is capable of binding to the *ccoN* promoter, which drives the expression of cytochrome *cbb*_3_ oxidase subunits. This study provides a new model of aerobic regulation of gene expression by RegA involving the formation of an intermolecular disulfide bond.

## 1. Introduction

Just as Ser/Thr/Tyr phosphorylation pathways are among the most common signaling pathways in eukaryotes, two-component systems (TCSs) constitute the most common signal transduction pathway in prokaryotes [1]. Receptor sensor kinases in TCSs sense various stimuli ranging from physical conditions such as light, temperature, redox state, osmolarity and concentration of chemicals such as nutrients and quorum signals [2]. TCSs control numerous cellular activities involved in metabolism, mobility and virulence [1].

In the purple non-sulfur bacterium *Rhodobacter capsulatus*, the RegB/RegA two-component system regulates a variety of physiological processes such as photosynthesis, hydrogen fixation, respiration, carbon fixation, nitrogen assimilation, denitrification, hydrogen uptake and aerotaxis [3]. Previous results indicate that RegB senses redox change through two sensing mechanisms. First, RegB has a ubiquinol/ubiquinone binding site located within a membrane-spanning region that monitors the redox state of the ubiquinol pool [4,5]. The binding of oxidized ubiquinone inhibits, while the binding of reduced ubiquinol stimulates, RegB kinase activity [4]. RegB has low affinity to both oxidized ubiquinone and reduced ubiquinol, allowing RegB to readily adjust its activity to the redox state of the ubiquinone pool, not unlike that of a rheostat [4,5]. The second level of RegB control involves direct inhibition of RegB kinase activity by dioxygen via oxidation of a conserved cytosolic cysteine residue (Cys265). The oxidation of Cys265 results in both the formation of a stable sulfenic acid derivative and disulfide bond formation that promotes stable tetramerization of RegB with both oxidized RedB derivatives exhibiting inactive kinase activity [6,7]. Collectively, these results support a long-standing model where RegB only undergoes autophosphorylation and subsequent transfer of the phosphoryl group to its cognate response regulator RegA under anaerobic conditions. Phosphorylated RegA then binds to DNA as a transcriptional factor, modulating the expression of genes involved in numerous anaerobic energy-generating and energy-utilizing processes [8,9]. While the described model has broad acceptance, RNA-seq data from our laboratory indicate that this model may be too simplistic as RegA is still capable of aerobically regulating ~47 genes, which is a condition in which RegA would not be phosphorylated by RegB [10]. We have therefore investigated whether RegA is capable of regulating gene expression under aerobic unphosphorylated conditions.

One clue for a possible alternative mechanism for controlling the activity of RegA in the absence of phosphorylation by RegB may be the transcriptional co-localization of *regB* and *regA* with *senC*. In species that contain the group 1 RegB/RegA system, *regA* and/or *regB* is invariably cotranscribed with *senC* [11,12]. SenC is a known copper-binding protein with proposed copper chaperone activity [13,14,15]. In *R. capsulatus*, SenC has a role in the assembly of copper containing *cbb*_3_ cytochrome oxidase [13,14,15]. However, SenC also has sequence similarity to a family of oxidoreductases that have thiol-disulfide bond forming activity [16]. Furthermore, group 1 RegA orthologs also contain an invariant conserved Cys (Cys156 in *R. capsulatus*) located immediately upstream of a DNA binding helix-turn-helix domain [12,17]. Thus, we explored whether the activity of RegA could be modulated by copper and/or the oxidation of Cys156. Our results indicate that RegA is capable of binding Cu^2+^ and that exposure of RegA-Cu^2+^ to O_2_ results in disulfide bond formation at Cys156, which stimulates dimerization and DNA binding.

## 2. Materials and Methods

### 2.1. Overexpression of RegA

The construction of a RegA overexpression vector follows the same method of construction of a RegA* overexpression vector as described in Du et al. (1998) [18] with the exception that the 95th residue of the RegA* codon was mutated from Alanine to Serine, which is the wild-type RegA codon. The resulting plasmid pET29CBD::*regA*, which also contains a chitin-binding domain fused to the C-terminal of RegA, was then transformed into the overexpression strain BL21(DE3) and grown overnight by shaking at 250 rpm at 37 °C. The overnight culture was then subcultured (1:50 ratio) into 1 L of Terrific Broth (TB) containing 50 µg/mL kanamycin. When the OD_600nm_ reached 0.5 to 0.8, the expression of RegA was induced by the addition of 0.4 mL 1M IPTG per liter culture and then shaken at 250 rpm at 37 °C for 4 h. Cells were then harvested by centrifuging at 10,000× *g* for 15 min. Cell pellets were stored at −80 °C prior to RegA purification.

### 2.2. Purification of RegA from E. coli

Frozen cell pellets from 2 L of RegA culture were resuspended in 50 mL lysis/wash buffer (20 mM Tris-HCl pH 8.0, 500 mM NaCl) and digested with 400 μL 2 mg/mL DNaseI via homogenization on ice and rotation at 4 °C for an hour. The cell lysate was then disrupted by three passages through an emulsifier and centrifuged at 17,210× *g* for 30 min. RegA containing an intein chitin-binding domain was purified by passing through a 10 mL chitin affinity column after equilibrating the column with 10 column volumes of lysis buffer, and then washed with another 10 column volumes of lysis/wash buffer. The intein chitin-binding domain was cleaved off RegA by purging the chitin affinity column with 3 column volumes of cleavage buffer (20 mM Tris-HCl pH 8.0, 500 mM NaCl, 30 mM DTT) and incubated at room temperature overnight. Tagless RegA was then eluted with 3 column volumes of elution buffer (20 mM Tris-HCl (pH 8.0), 500 mM NaCl). Finally, reduced RegA monomer was treated with gel filtration chromatography using a Superose 12 column equilibrated with 20 mM Tris-HCl pH 8.0, 250 mM NaCl, 5 mM TCEP, 10 mM EDTA and 10% glycerol. Finally, RegA was concentrated to 2 to 3 mg/mL and stored at −80 °C.

### 2.3. Loading RegA with Metal through Buffer Exchange

An aliquot of purified RegA was concentrated to 100 μL in a 500 μL centrifugal filter (Amicon, Burlington, MA, USA) with the 10 kDa cutoff by centrifuging at 17,000× *g* for 8 min. RegA was then buffer exchanged into 20 mM Tris-HCl pH 8.0 250 mM NaCl by repeating the steps of adding 400 μL fresh buffer and centrifuging at 17,000× *g* five times with final centrifugation run until the sample volume reached 100 μL. The concentration of RegA was then measured using the Bradford method. The RegA sample was then anaerobically loaded with metals by incubating for 5 min with 400 μL 20 mM Tris-HCl pH 8.0 250 mM NaCl containing 3× metal to 1× RegA’s concentration. CuCl and CuSO_4_ solutions were prepared inside the anaerobic chamber using degassed ddH_2_O for copper salt preparation. The protein buffer (20 mM Tris-HCl pH 8.0, 500 mM NaCl) used for buffer exchange was also degassed and purged with nitrogen before being brought into the anaerobic chamber. Excessive metal not bound to RegA was then removed from the buffer by repeating Amicon filter centrifugation eight times (maximum speed until ~100 μL concentrated sample was left) with each step involving the addition of 400 μL fresh buffer.

### 2.4. ICP-MS

The concentrations of protein were measured before analysis. At least 20 μL protein of 20 μM or 500 μL buffer was required to conduct inductive coupled plasma mass spectrometry (ICP-MS). Protein samples of RegA were diluted 100-fold with 2.5% HNO_3_ (Sigma) into a final volume of 3 mL, while using Pure Plus Internal Standard Mix (100 ppb, PerkinElmer, Waltham, MA, USA) as an internal standard. Using hydrogen (^55^Mn, ^56^Fe, ^59^Co, ^60^Ni detection) or helium (^24^Mg, ^63^Cu, ^66^Zn detection) as collision gases to remove possible interferences with the internal reference ^45^Sc or ^72^Ge, samples were analyzed with an Agilent 8800 QQQ ICP-MS device. Based on standard curves of all analyzed metals generated with Pure Plus Multi-Element Calibration Standard 3 (0.5–100 ppb, PerkinElmer), metal concentrations of protein or buffer samples were calculated.

### 2.5. ESI-MS

RegA were incubated with a gradient concentration of CuSO_4_ in one-fold, three-fold, five-fold and ten-fold of RegA concentration, respectively. Measures of 30 μL of these Cu^2+^ treated RegA samples, apo-RegA and buffer were separately injected and sealed in glass vials and brought out of the anaerobic chamber. The electrospray ionization mass spectrometry (ESI-MS) spectra of the samples were generated using an LC (C_4_ reverse phase)-MS (Synapt G2S HDMS) instrument and analyzed with Mass Lynx v4.1.

### 2.6. LC-MS/MS

Gel bands were diced into 1 mm cubes and incubated for 45 min at 57 °C. To maintain potential disulfide bonds, the samples were neither reduced nor alkylated. A solution containing 1 μg trypsin, in 25 mM ammonium bicarbonate was added and the samples were digested for 12 h at 37 °C. The resulting peptides were desalted using a ZipTip (Millipore, Billerica, MA, USA). The samples were dried down and injected into an Eksigent HPLC coupled to an LTQ Orbitrap XL (Thermo Fischer Scientific, Waltham, MA, USA). The peptides were separated using a 75 micron, 15 cm column packed in-house with C18 resin (Michrom Bioresources, Auburn, CA, USA) at a flow rate of 300 nl/min. A one-hour gradient was run from Buffer A (2% acetonitrile, 0.1% formic acid) to 60% Buffer B (100% acetonitrile, 0.1% formic acid). The Orbitrap was configured to acquire a survey scan over the mass range 300–2000 at a resolution of 30,000. This was followed by collision-induced dissociation mass spectrometry (CID MS/MS) on the top-five most-intense precursor ions above a threshold of 1000 counts.

The resulting MS/MS peaklists were searched against the Uniprot *Rhodobacter capsulatus* database using Protein Prospector (v5.10.14). Acetylation of the protein amino terminus, oxidation of methionine and pyroglutamine formation of peptide amino terminal glutamine and disulfide bond formation were set as variable modifications. A mass tolerance of 20 ppm was used for precursor ions and 0.6 Da was used for fragment ions. Peptide expectation values were set to <0.05.

### 2.7. DNase I FootPrinting Assay

DNase I footprint analysis was performed as described by Willett et al. [19]. Briefly, a 299 bp DNA fragment containing the promoter region of *ccoN* was PCR amplified using a 6-FAM labeled forward primer and a HEX labeled reverse primer. A 50 nM DNA probe was incubated at room temperature for 15 min with a gradient concentration of purified proteins in 20 mM Tris-HCl pH 8.0, 2 mM MgCl_2_, 0.5 mM CaCl_2_, 0.1 mg/mL BSA of a total volume of 20 μL and then digested with 5 μL of DNaseI for another 15 min at room temperature. The reactions were quenched by the addition of 25 μL 0.5 M EDTA pH 8.0. The digested fragments were recovered using a Min Elute PCR purification kit (Qiagen, Hilden, Germany) and then eluted with 15 μL elution buffer. The samples were detected with a 3730 DNA Analyzer (Applied Biosystem, Waltham, MA, USA) using 500 LIZ^TM^ Size Standard and eventually analyzed with the Peak Scanner Software v2.0.

## 3. Results

### 3.1. RegA Binds Copper Anaerobically

**Figure 1:**
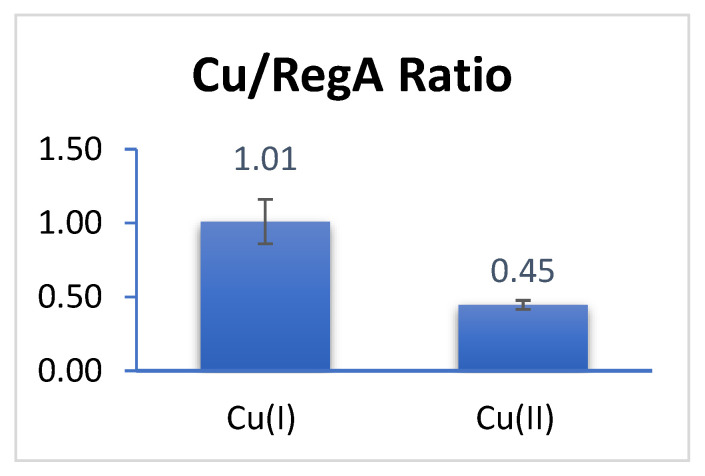
Ratio of concentration of copper ion over RegA anaerobically.

We first addressed whether RegA can bind copper in the Cu^1+^ or Cu^2+^ states. Since Cu^1+^ is only stable in the absence of oxygen (oxygen oxidizes Cu^1+^ to Cu^2+^), we assayed the ability of RegA to bind these two redox states of Cu under anaerobic conditions. For this analysis, we anaerobically exposed RegA to threefold molar excess of either Cu^1+^ or Cu^2+^ followed by removal of unbound Cu using centrifugal filtration mediated buffer exchange in an anaerobic chamber. After unbound Cu^1+^ and Cu^2+^ were removed, we measured the concentration of copper that remained bound to RegA using inductively coupled plasma mass spectrometry (ICP-MS). As shown in Figure 1, RegA anaerobically binds approximately stoichiometric amount of Cu^1+^, which is indicated by a copper ion over RegA monomer ratio of 1.02 ± 0.21. In contrast, the ratio of Cu^2+^ over RegA is very close to 0.5 (0.45 ± 0.03), suggesting that two RegA’s bind to one molecule of Cu^2+^ anaerobically. Taken together, these results indicate that RegA is indeed capable of binding copper.

### 3.2. Cu^2+^ Promotes the Dimerization of RegA Aerobically

**Figure 2:**
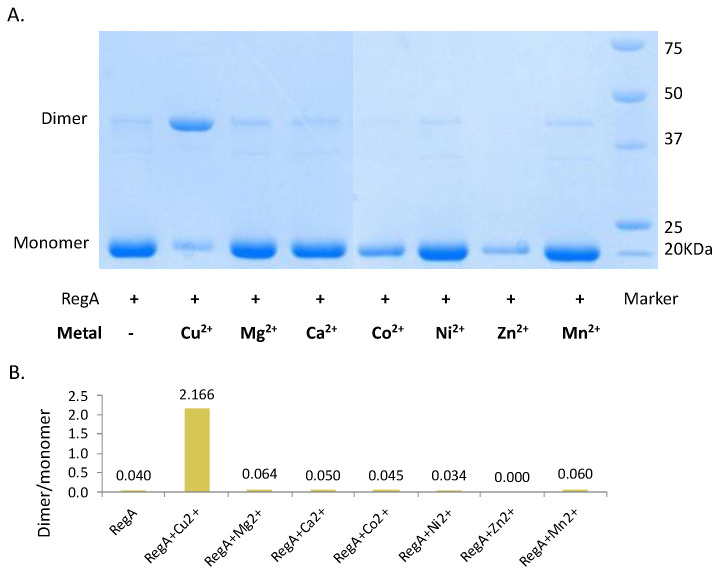
Cu^2+^ indispensably promotes the dimerization of RegA. (**A**) Non-reducing SDS-PAGE gel of apo-RegA and RegA treated with divalent metals. + denotes addition of RegA protein whereas –denotes where metal is not added. (**B**) Histogram of RegA dimer:monomer ratios determined by image J.

**Figure 3:**
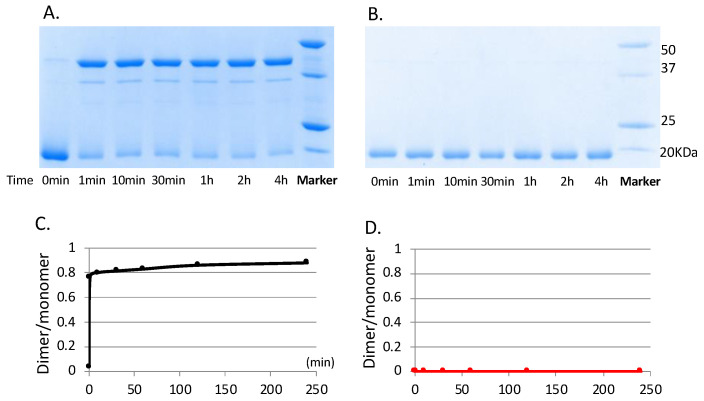
Copper catalyzes the dimerization of RegA likely with the help of oxygen. (**A**) Non-reducing SDS-PAGE gel of RegA treated with Cu^2+^ in the air after different time periods. (**B**) Non-reducing SDS-PAGE gel of RegA in the absence of Cu^2+^ in the air after different time periods. (**C**) Dimer:monomer ratio of RegA treated with Cu^2+^ in the air after different time periods. (**D**) Dimer:monomer ratio of RegA in the absence of Cu^2+^ in the air after different time periods. The dimer:monomer ratio was calculated based on the intensity of bands quantified through Image J v1.51.

Since Cu^2+^ binds reduced RegA in a 0.5:1 ratio, we next investigated whether Cu^2+^ was capable of stimulating dimerization of RegA. For this analysis, monomeric RegA was loaded with Cu^2+^ via buffer exchange aerobically and immediately run through a non-reducing SDS-PAGE gel with no DTT added to the samples. As shown in Figure 2, the addition of Cu^2+^ indeed promotes the dimerization of RegA. As observed from the non-reducing SDS-PAGE gel in Figure 3A, Cu^2+^ mediated dimerization of RegA is also quite rapid, with the majority of RegA dimerized after only one minute of Cu^2+^ treatment in the presence of air. In contrast, exposure of RegA to air in the absence of Cu^2+^ did not lead to any significant dimerization even after 4 h of exposure, confirming that Cu^2+^ in the presence of oxygen serves a critical role in mediating the dimerization of RegA (Figure 3B).

To determine whether Cu^2+^ uniquely plays a role in promoting dimerization of RegA, we also treated RegA with six other divalent metals including Mg^2+^, Ca^2+^, Co^2+^, Ni^2+^, Zn^2+^ and Mn^2+^ following the same protocol. The non-reducing SDS-PAGE gel results in Figure 2 show that none of the other six divalent metals tested stimulated RegA dimerization, indicating that only Cu^2+^ is capable of promoting dimerization of RegA.

### 3.3. RegA Dimerization Requires Both Cu^2+^ and Molecular Oxygen

**Figure 4:**
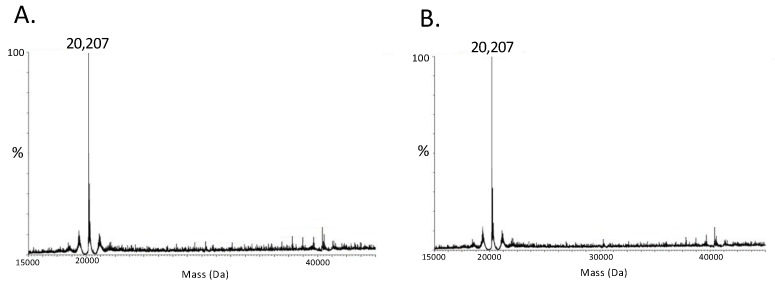
ESI-MS spectrum of RegA in the presence or absence of Cu^2+^ anaerobically. (**A**) RegA molecular weight determination by ESI-MS (20,207 daltons) in the presence of Cu^2+^ without oxygen. (**B**) RegA molecular weight determination by ESI-MS (20,207 daltons) in the absence of Cu^2+^ without oxygen.

We next addressed whether molecular oxygen is a requirement for Cu^2+^ dimerization of RegA. For this analysis, we tested whether Cu^2+^ can promote the dimerization of RegA under anaerobic conditions by treating RegA monomers with Cu^2+^ in an anaerobic chamber with degassed buffers for 10 min. The reactions were quenched with EDTA (or passed through filters) to remove Cu^2+^ prior to analysis of the monomer/dimer state of RegA using electrospray ionization mass spectrometry (ESI-MS). As shown in Figure 4, the RegA anaerobically treated with Cu^2+^ and a monomer control without Cu^2+^ treatment both showed identical ESI-MS profiles exhibiting a monomer molecular weight of 20,207 daltons. This indicates that both Cu^2+^ and atmospheric levels of molecular oxygen are necessary to promote the dimerization of RegA.

**Figure 5:**
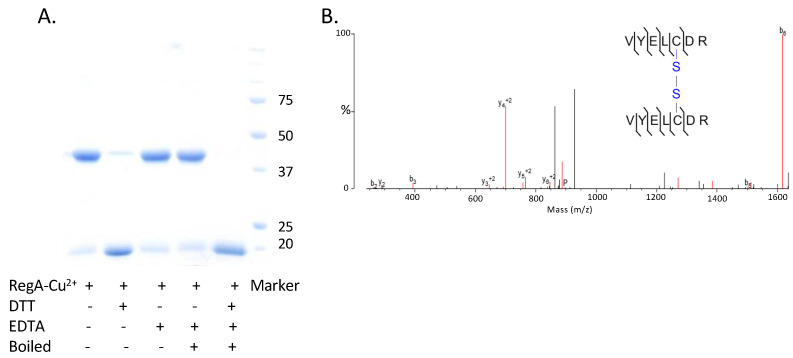
Cu^2+^ can promote RegA to dimerize with the formation of a disulfide bond in vitro. (**A**) Non-reducing SDS-PAGE gel of Cu^2+^ treated RegA dimer treated with EDTA or DTT. (**B**) Disulfide bond formation in Cu^2+^treated RegA dimer observed in LC-MS/MS.

We next addressed whether the dimer formed by Cu^2+^ treated RegA involves a covalent disulfide bond or instead is a non-covalent interaction. For this analysis, Cu^2+^ + O_2_ mediated RegA dimer was isolated away from monomer RegA by gel filtration chromatography and then treated with either DTT or EDTA alone or both. As is indicated in Figure 5, the addition of excess EDTA, which would remove Cu^2+^, did not affect dimerization. This is contrasted by the conversion of RegA dimers to its monomeric form by the addition DTT, which would indicate that Cu^2+^ likely stimulates disulfide bond formation. For confirmation, we also undertook liquid chromatography–tandem mass spectrometry (LC-MS/MS) of trypsin-digested Cu^2+^-formed RegA dimer, which showed the presence of a peptide fragment VYELCDR containing an intermolecular disulfide bond at Cys156 (Figure 5B). Interestingly, RegA only has one cysteine residue at the 156th position, with this cysteine residue conserved among all the α-proteobacteria that contain RegA homologs. Finally, analysis of the presence of Cu in the isolated RegA dimers with ICP-MS shows no significant amounts of Cu present in covalently linked dimerized RegA.

### 3.4. Covalently Dimerized RegA Is Capable of Binding DNA

Phosphorylation of response regulators by cognate sensor kinases is known to stimulate dimerization, which facilitates DNA binding. Given that unphosphorylated RegA monomers have low DNA binding activity [20], we next addressed whether RegA dimers formed by disulfide bond formation are also capable of binding to DNA. For this analysis, we performed DNase I footprint assays with Cu^2+^ stimulated RegA disulfide dimer to the *ccoN* promoter region that controls cytochrome *cbb*_3_ oxidase expression. This promoter was chosen for analysis based on previous transcriptomic analyses, which showed that RegA regulates the expression of the *ccoN* promoter under aerobic conditions when RegB kinase activity is suppressed [3,10].

**Figure 6:**
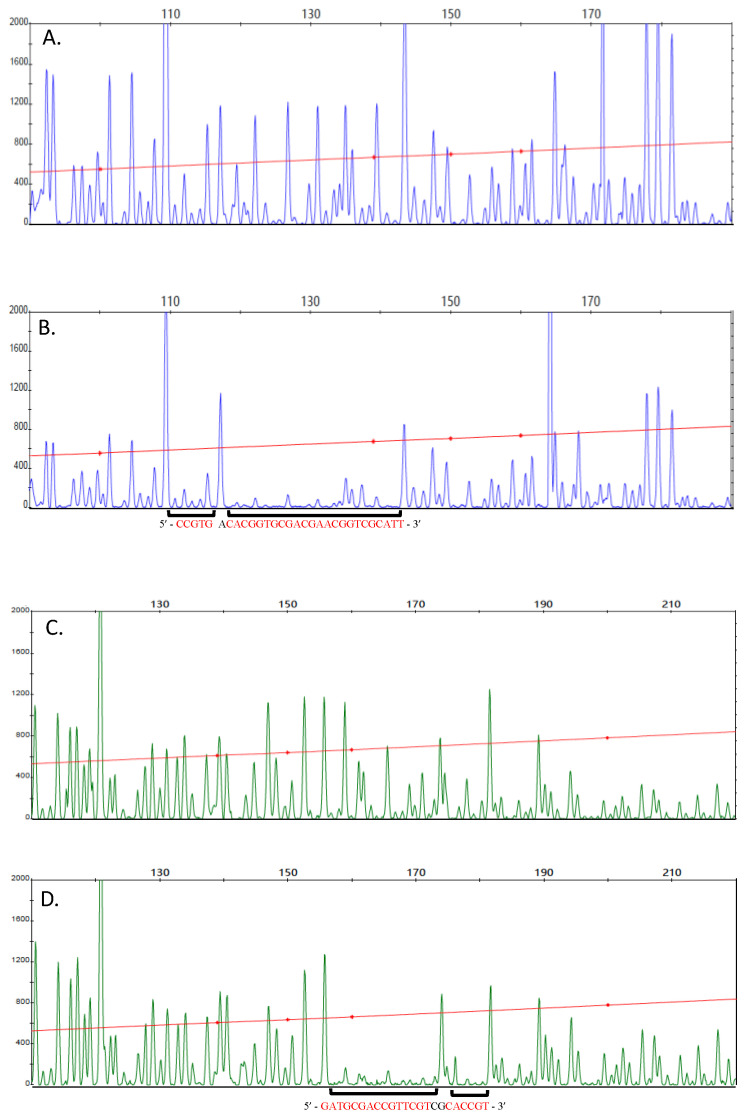
DNaseI footprinting of Cu^2+^-treated RegA dimer to the *ccoN* promoter. (**A**) DNase I digestion of the *ccoN* promoter top strand without RegA. (**B**) DNase I digestion of the *ccoN* promoter top strand with 2 µM Cu^2+^ treated unphosphorylated RegA dimer. (**C**) DNase I digestion of the *ccoN* promoter bottom strand without RegA. (**D**) DNase I digestion of the *ccoN* promoter bottom strand with 2 µM Cu^2+^ treated unphosphorylated RegA dimer. Areas of RegA protection are highlighted with a black bar.

Footprint results shown in Figure 6 demonstrate that covalently dimerized unphosphorylated RegA can bind to the *ccoN* promoter region, as exhibited by excellent DNase I peak suppression with 2 µM of dimerized RegA. Two binding sites highlighted in red are readily observed on both the forward and the reverse strands (Figure 6B,D, respectively). The two forward strand protection sites observed with Cu^2+^ treated RegA dimers (−80 bp to −76 bp and −74 bp to −49 bp upstream of the transcription initiation site, respectively) are at the same location as previously reported with a constitutively dimerized and active RegA variant called RegA* [3]. On the reverse strand, RegA dimers also bind at two binding sites (−74 bp to −69 bp and −66 bp to −52 bp upstream of the transcription initiation site), which are just slightly narrower than the binding site observed with RegA* dimers (−74 bp to −49 bp) [3]. Footprint analysis with varying amounts of dimerized RegA shows that Cu^2+^ stimulated RegA dimer binds to the ccoN promoter with an apparent Kd of 1 μM ~ 4-fold lower than that observed with RegA* (0.25 μM) [3]. These results demonstrate that covalently dimerized unphosphorylated RegA indeed binds to the *ccoN* promoter at just a slightly lower affinity than phosphorylated noncovalent dimers do.

## 4. Discussion

The response regulator paradigm is that phosphorylation of a response regulator by its cognate histidine kinase promoted dimerization and subsequent DNA binding activity. This study shows that RegA from *R. capsulatus* binds copper and that bound Cu^2+^ in the presence of O_2_ leads to intermolecular disulfide bond formation. Covalently dimerized RegA is capable of binding DNA at sub μM concentrations, which likely provides RegA the ability to aerobically regulate gene expression in the absence of phosphorylation. 

The formation of disulfide bonds has been adopted by several transcription factors to control their DNA binding activities in response to redox changes. Some well-characterized examples are OxyR, which, in response to hydrogen peroxide or S-nitrosothiols, induces the expression of over ten genes encoding proteins [21] against oxidative and nitrosative stress; and *oxyS*, a gene that encodes a nontranslated RNA involved in DNA repair. In both cases, exposure to oxygen stimulates disulfide bond oxidation that affects DNA binding activity [22]. In *R. capsulatus*, CrtJ is an aerobic repressor of photosystem genes that has regulatory cysteins that are oxidized in the presence of molecular oxygen [23,24,25]. Besides the use of disulfide bond formation, other transcription factors have utilized changes in the redox state of iron to sense the presence or absence of oxygen. For example, FNR, a global regulator of over a hundred genes in *E. coli*, contains a [4Fe-4S] iron–sulfur cluster that disassembles under aerobic conditions, which disrupts dimerization and subsequent binding to DNA [21,26,27]. In the case of RegA, not only does copper itself become oxidized by oxygen from Cu^+1^ to Cu^+2^, but, in addition, the presence of both Cu^2+^ and oxygen are needed for disulfide bond formation. In some respects, this may be regarded as a hybrid of the above-described mechanisms of redox sensing.

### 4.1. A Model Describing How Copper and Oxygen Promote RegA Disulfide Bond Formation

**Figure 7:**
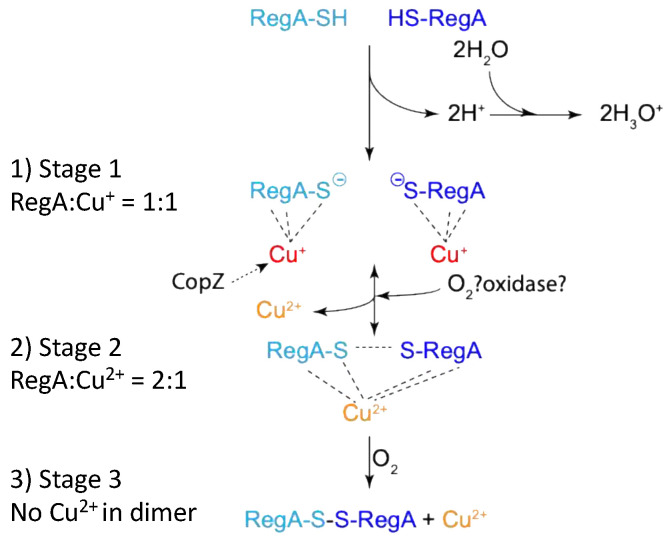
Proposed model of the mechanism of how Cu^2+^ promotes the dimerization of RegA in vivo.

Taking all of our results into consideration, we can propose a model of how Cu^2+^ and oxygen likely promote covalent dimerization of RegA as follows (Figure 7): (i) anaerobically, RegA monomer binds Cu^1+^, possibly by accepting Cu^1+^ transfer from an intracellular copper chaperone such as from CopZ [28] or other copper chaperones. (ii) When the environment changes from anaerobic to aerobic conditions, oxygen oxidizes RegA bound Cu^1+^ into Cu^2+^. Cu^2+^ with the help of O_2_ then promotes subsequent disulfide bond formation. (iii) Disulfide bond dimerized RegA then binds to a subset of promoters.

**Figure 8:**
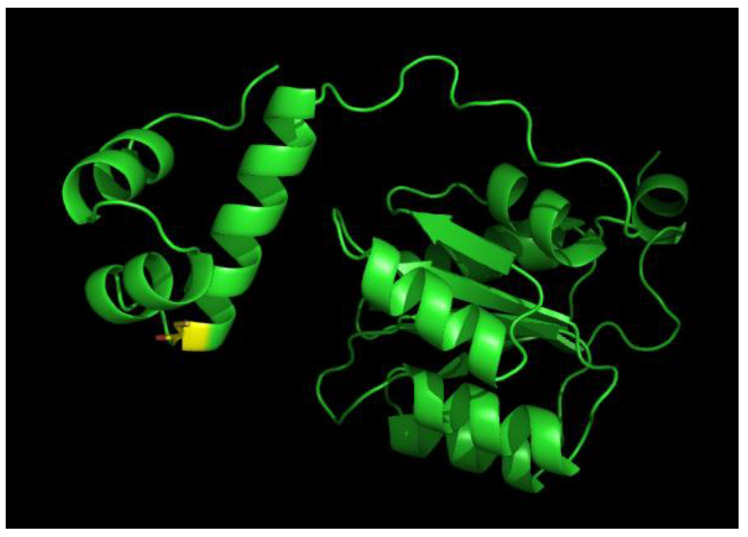
RegA tertiary structure as predicted from the DeepMind released artificial intelligence program AlphaFold. The DNA binding helix-turn-helix domain is on the left and the receiver domain is on the right. Cys156 that undergoes disulfide bond formation is highlighted in yellow.

Recently, Alphabet’s DeepMind released the artificial intelligence program AlphaFold that predicts a protein’s tertiary structures based on a protein’s primary sequence [29]. Analysis of a putative RegA structure derived from AlphaFold shows two well-defined domains (Figure 8). The largest right-side domain in Figure 8 is very similar to response regulator receiver domains that have previously been solved by X-ray crystallography [1]. This phosphorylatable receiver domain is linked by a long, flexible linker region containing five prolines to a DNA binding domain shown on the left. The two smallest left-most helices exhibit a high degree of structural similarity to the RegA helix-turn-helix DNA binding helices as previously solved by NMR [17]. The larger helix after the flexible linker region just before the two small DNA binding helices contains the universally conserved Cys156 as shown in yellow. Given the flexibility of this domain from the receiver domain, this Cys should be available for oxidation into an intermolecular disulfide by molecular oxygen. We suspect that the role of Cu^2+^ may be a structural role where it binds and holds two RegA monomers in such a position that this helix from two RegA’s are positioned in close proximity, thereby allowing oxygen to effectively promote disulfide bond formation.

Due to the capability of copper ions to undergo reversible oxidation of Cu^1+^ to Cu^2+^, copper acts as an essential cofactor for several Cu metalloenzymes such as cytochrome *cbb*_3_ oxidase encoded by the *ccoN* operon that RegA aerobically and anaerobically regulates and superoxide dismutase [30]. However, copper unbound to protein is also quite toxic. For example, Cu^1+^ tends to mediate the generation of radical oxygen species (ROS) through Fenton reaction [31,32], while Cu^2+^ can nucleate protein aggregation, leading to amyloid formation in mammals [30]. Hence, the transportation and distribution of copper is precisely regulated [33]. Apart from copper importers and exporters, copper ions are often delivered to copper-required proteins through copper chaperones [34] in the form of Cu^1+^. It is therefore plausible that one or several copper chaperones may directly interact and transfer copper to RegA. SenC is unlikely to itself deliver copper to RegA as SenC’s copper binding domain is located in the periplasm [14,15]. Besides SenC [13,14,15], the identified copper chaperones in *Rhodobacter* include a Cu^1+^ chaperone Cox11 [35] in *Rhodobacter sphaeroides*, a PCuAC-like periplasmic chaperone PccA [15], and a recently identified intracellular Cu^1+^ chaperone CopZ [28], in *Rhodobacter capsulatus*. Since RegA is an intracellular protein, and the copper-binding site of SenC is in the periplasm [14], we suspect that cytoplasmic CopZ most likely transfers Cu^1+^ to RegA in *R. capsulatus*.

### 4.2. Both Aerobic and Anaerobic Conditions Control RegA Activity

**Figure 9:**
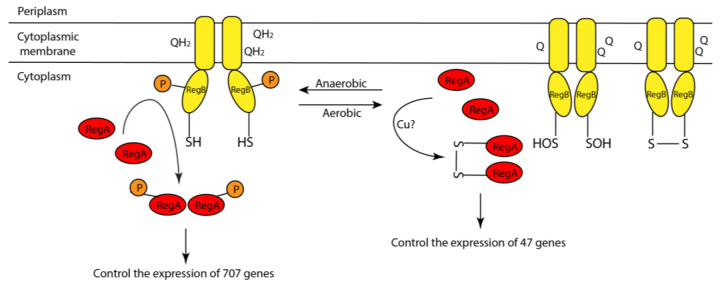
Model of RegB/RegA two-component system in sensing redox changes and regulating gene expressions anaerobically (**left**) and hypothetic model of how RegA regulates gene expressions aerobically (**right**).

Most previous studies have focused on the role of RegA, and its cognate sensor kinase RegB, in controlling anaerobic gene expression [8,9]. These studies demonstrated that the phosphorylation activity of RegB is controlled by both redox changes in the ubiquinone pool as well as by oxidation of a conserved cystine (Figure 9) [4,5,6,7]. RegB has six membrane-spanning helices, three of which are known to be involved in binding both oxidized and reduced ubiquinone [4,5,36]. When bound to oxidized ubiquinone, the kinase activity is low, whereas when bound to reduced ubiquinol, the kinase activity is high [4] (Figure 9). Additionally, there is a redox-active Cys in the four-helix bundle that links the membrane-spanning domain to the kinase domain [6,7]. In the presence of oxygen, this Cys undergoes oxidation either to a sulfonic acid derivative or to a disulfide, either of which inhibits kinase activity (Figure 9) [6,7].

Evidence that RegA itself may undergo redox control was recently revealed when it was observed that RegA controls the expression of ~47 genes under aerobic growth conditions when RegB kinase activity is inhibited [10]. Interestingly, all 47 genes that are aerobically regulated by unphosphorylated RegA also belong to the group of 707 genes that are anaerobically regulated by phosphorylated RegA [10]. We suspect that there must be a subtle difference between aerobic disulfide dimerized unphosphorylated RegA and anaerobic phosphorylated RegA that differentiates which promoters that these two variations of RegA function with. For example, the binding of Cu^2+^ and O_2_ dimerized RegA to the *ccoN* promoter appears to be slightly lower than that observed with the constitutively active variant RegA*. Even such a subtle difference in binding affinity may have significant effects on the promoters that RegA controls. There could also be differences by which these two differently dimerized variants interact with RNA polymerase. Further additional comparative studies will have to be undertaken to reveal how promoter activity is differentially regulated by these variants.

## Data Availability

Not applicable.
